# The Genetics of Schizophrenia

**DOI:** 10.1371/journal.pmed.0020212

**Published:** 2005-07-26

**Authors:** Patrick F Sullivan

## Abstract

Research into the etiology of schizophrenia, particularly the possible genetic basis, has never been as interesting and as provocative as in the past three years. Sullivan looks critically at the key research.

Research into the etiology of schizophrenia has never been as interesting or as provocative as in the past three years. There has been progress on several fronts, but particularly regarding the molecular genetics of this complex disorder of mind and brain. At the same time, a number of critically important and unresolved issues remain that qualify the ultimate clinical and scientific validity of the results. However, the recent progress in this historically difficult area of inquiry does not seem to be widely appreciated. The purpose of this article is to provide a high-level review of progress, its limitations, and the implications for clinical research and clinical practice.

The public health importance of schizophrenia is clear. The median lifetime prevalence of schizophrenia is 0.7–0.8% [1], with onset typically ranging from adolescence to early adulthood and a course of illness typified by exacerbations, remissions, and substantial residual symptoms and functional impairment [2]. Morbidity is substantial, and schizophrenia ranks ninth in global burden of illness [3]. In addition, schizophrenia is often comorbid with drug dependence (principally alcohol, nicotine, cannabis, and cocaine) and important medical conditions (obesity, Type 2 diabetes mellitus) [4]. Mortality due to natural and unnatural causes is considerable, and the projected lifespan for individuals with schizophrenia is some 15 years less than the general population [5]. The personal, familial, and societal costs of schizophrenia are enormous.

## Etiological Clues

**Figure 1:**
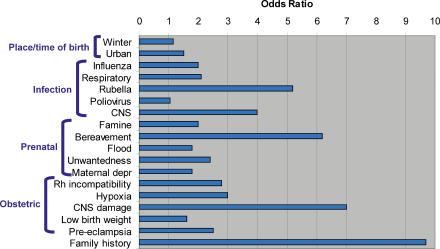
Comparison of a Selected Set of Relatively Well-Established Risk Factors for Schizophrenia, Focusing Mainly on Pre- and Antenatal Factors [6] (abbreviations: CNS, central nervous system; depr, depression; Rh, Rhesus)

A substantial body of epidemiological research has established a set of risk factors for schizophrenia. A subset of this work is summarized in Figure 1. Of a large set of pre- and antenatal risk factors [6], having a first-degree relative with schizophrenia is associated with an odds ratio of almost ten. The general impact of some of the risk factors in Figure 1 remains uncertain, and, additionally, migrant status, urban residence, cannabis use, and biological sex are supported as risk factors for schizophrenia. Although the attributable risk of some of these risk factors may be greater (e.g., place and season of birth) [7], the size of the odds ratio for family history suggests that searching for the familial determinants of schizophrenia is rational for etiological research.

## Unpacking the Family History Risk Factor

Studies of families, adoptees, and twins have been widely used to attempt to understand the relative contributions of genetic and environmental effects upon risk for schizophrenia. These “old genetics” approaches use phenotypic resemblance of relatives as an indirect means by which to infer the roles of genes and environment. There are many important assumptions and methodological issues with these studies [8]; however, genetic epidemiological studies of schizophrenia have yielded a remarkably consistent set of findings, as summarized in Table 1 [9, 10].

**Table 1:**
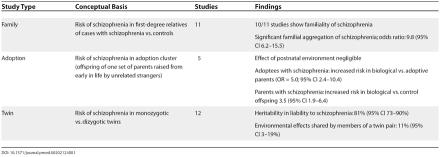
Summary of Studies of the Genetic Epidemiology of Schizophrenia

To summarize this literature briefly, schizophrenia is familial, or “runs” in families. Both adoption and twin studies indicate that the familiality of schizophrenia is due mainly to genetic effects. Twin studies suggest the relevance of small but significant shared environmental influences that are likely prenatal in origin. Thus, schizophrenia is best viewed as a complex trait resulting from both genetic and environmental etiological influences. These results are only broadly informative, as they provide no information about the location of the genes or the identity of the environmental factors that predispose or protect against schizophrenia. Searching for genetic influences that mediate vulnerability to schizophrenia is rational, given the larger overall effect size and lesser error of measurement in comparison to typical assessments of environmental effects. Note that high heritability is no guarantee of success in efforts to identify candidate genes.

## Genomewide Linkage Studies of Schizophrenia

**Figure 2:**
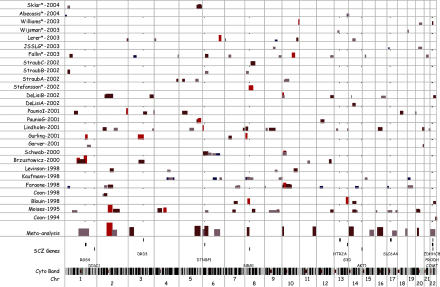
Summary of Genomewide Linkage Studies of Schizophrenia The *x*-axis shows the location on the genome, from the telomere of the short arm of Chromosome 1 to the telomere of the long arm of Chromosome 22 (bottom row) along with 303 band chromosomal staining on the second-to-bottom row. The *y*-axis shows the 27 primary samples that reported first-stage genome scans for schizophrenia (i.e., excluding fine-mapping or partial reports) along with the results of a meta-analysis including most of the primary samples [12] (studies not included are shown with asterisks). Within each row, the height and color of the bars are proportional to the –log_10_(*P*-value), and the width of the bar shows the genomic location implicated by a particular sample. A selected set of candidate genes for schizophrenia are also shown. All genomic locations are per the hg16 build (http://genome.ucsc.edu). The physical positions of an inclusive set of the markers showing the best findings in the primary samples were plotted (assuming a confidence interval of ± 10 cM or, if mapping was uncertain, ± 10 megabases; seven markers from the primary samples did not map).

Modern genotyping technologies and statistical analyses have enabled the discovery of genetic loci related to the etiology of many complex traits [11], such as Type 2 diabetes mellitus, obesity, and Alzheimer's disease. These “discovery science” approaches have been applied to schizophrenia, and are summarized in Figure 2. The 27 samples shown here included from one to 294 multiplex pedigrees (see Glossary) (median 34) containing 32 to 669 (median 101) individuals affected with a narrow definition of schizophrenia. There were 310 to 950 (median 392) genetic markers in the first-stage genome scans.

“Hard” replication—implication of the same markers, alleles, and haplotypes in the majority of samples—is elusive. It is evident from Figure 2 that these studies are inconsistent, and no genomic region was implicated in more than four of the 27 samples. The Lewis et al. meta-analysis [12] included most of the studies in Figure 2 and found that one region on Chromosome 2 was stringently significant and several additional regions neared significance. Our focus on first-stage genome scans does not adequately capture the evidence supporting replication for certain regions (e.g., 6p) [13–18]. However, there appears to be “soft” replication across studies.

It is unlikely that all of these linkage findings are true. The regions suggested by the Lewis et al. meta-analysis implicate more than 3,000 genes (18% of all known genes). For the 27 samples in Figure 2, the percentages of all known genes implicated by 0, 1, 2, 3, and 4 linkage studies were 42%, 35%, 14%, 6%, and 3%, respectively. This crude summation suggests that linkage analysis is an imprecise tool—implausibly large numbers of genes are implicated and few genes are consistently identified in more than a small subset of studies.

There are several potential reasons why clear-cut or “hard” replication was not found. With respect to the teams that conducted these enormously effortful studies, it is possible that no study possessed sufficient statistical power to detect the subtle genetic effects suspected for schizophrenia. For example, it would require 4,900 pedigrees to have 80% power to detect a locus accounting for 5% of variance in liability to schizophrenia at α = 0.001. These calculations make highly optimistic assumptions, and less favorable assumptions can lead to sample size requirements above 50,000 sibling pairs. For comparison, the total number of pedigrees in Figure 2 is less than 2,000.

In addition, it is possible that etiological heterogeneity (different combinations of genetic and environmental causes between samples) and technical differences (different ascertainment, assessment, genotyping, and statistical analysis between samples) contributed; however, their impact is uncertain, whereas insufficient power is clear. If correct, the implication is that Figure 2 contains a mix of true and false positive findings.

## Association Studies of Schizophrenia

Schizophrenia—like most other complex traits in biomedicine—has had a large number of genetic case-control association studies [19]. Although research practice is changing, interpretation of many studies is hindered by small sample sizes and a tendency to genotype a single genetic marker of the hundreds that might be available in a gene. For example, a widely studied functional genetic marker in *COMT* (rs4680) is probably not associated with schizophrenia [20], but nearby genetic markers assessed in a minority of studies may be [21].

However, as discussed in the next section, a number of methodologically adequate association studies of schizophrenia appear to support the role of several candidate genes in the etiology of schizophrenia. Similar to the linkage study data, “hard” replication remains elusive.

## Synthesis

Despite the limitations of the accumulated linkage and association studies, there are good suggestions that these studies have identified plausible candidate genes for schizophrenia. Table 2 summarizes the evidence in support of a set of possible candidate genes for schizophrenia. Reports supporting the role of many of these genes have appeared in top-tier international journals known for rigorous peer review. The evidence for several genes is encouraging but currently insufficient to declare any a clear-cut cause of schizophrenia.

**Table 2:**
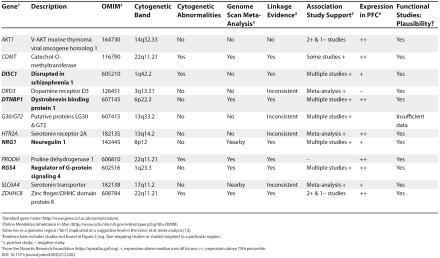
Evidence Supporting 12 Potential Candidate Genes for Schizophrenia

^1^Standard gene name (http://www.gene.ucl.ac.uk/nomenclature).

^2^Online Mendelian Inheritance in Man (http://www.ncbi.nlm.nih.gov/entrez/query.fcgi?db=OMIM).

^3^Gene lies in a genomic region (“bin”) implicated at a suggestive level in the Lewis et al. meta-analysis [12].

^4^Evidence here includes studies not found in Figure 2 (e.g., fine-mapping studies or studies targeted to a particular region).

^5^+, positive study; −, negative study.

^6^From the Novartis Research Foundation (http://symatlas.gnf.org). +, expression above median over all tissues; ++, expression above 75th percentile.

The accumulated data provide particular support for *DISC1*, *DTNBP1*, *NRG1*, and *RGS4*. Each of these genes has received support from multiple lines of evidence with imperfect consistency: 1) The case for each of these as a candidate gene for schizophrenia is supported by linkage studies; 2) The preponderance of association study findings provides further support; 3) mRNA from each gene is expressed in the prefrontal cortex as well as in other areas of the brain; and 4) Additional neurobiological data link the functions of these genes to biological processes thought to be related to schizophrenia. For example, *DISC1* modulates neurite outgrowth, there is an extensive literature on the involvement of *NRG1* in the development of the CNS, and *RGS4* may modulate intracellular signaling for many G-protein-coupled receptors. Moreover, *DTNBP1* and *RGS4* have been reported to be differentially expressed in postmortem brain samples of individuals with schizophrenia.

This encouraging summation of work in progress masks a critical issue—the lack or consistent replication for the same markers and haplotypes across studies. The literature supports the contention that genetic variation in these genes is associated with schizophrenia, but it lacks impressive consistency in the precise genetic regions and alleles implicated. In contrast, association studies of other complex human genetic diseases have produced unambiguous, consistent, and clear-cut (“hard”) replication. For example, 1) in Type 1 diabetes mellitus, the bulk of both the linkage and association data implicate the *HLA* region and *INS* [22]; 2) for Type 2 diabetes mellitus, there are a number of findings in the literature where the association evidence appears to be consistent and compelling (*CAPN10*, *KCNJ11*, and *PPARG*)—the data indicate that the same marker allele is significantly associated and has an effect size of similar direction and magnitude [22] (the linkage data are less congruent, probably due to power considerations); and 3) for age-related macular degeneration, at least five studies show highly significant association with the same *CFH* Y402H polymorphism [23–27] in a region strongly implicated by multiple linkage studies. For these findings, the data are highly compelling and consistent and provide a solid foundation for the next generation of studies to investigate the mechanisms of the gene–phenotype connection. Power/type 2 error appears to be a major factor—if the genetic effect is sufficiently large (*HLA* in Type 1 diabetes mellitus or *CFH* in age-related macular degeneration)—or, if the sample size is large, then there appears to be a greater chance of “hard” replication.

At present, the data for schizophrenia are confusing, and there are two broad possibilities. The first possibility is that the current findings for some of the best current genes are true. This implies that the genetics of schizophrenia are different from other complex traits in the existence of very high degrees of etiological heterogeneity: schizophrenia is hyper-complex, and we need to invoke more complicated genetic models than other biomedical disorders. The alternative possibility is that the current findings are clouded by Type 1 and Type 2 error. Schizophrenia is similar to other complex traits: it is possible that there are kernels of wheat, but it is highly likely that there is a lot of chaff. At present, the second and more parsimonious possibility has not been rigorously excluded. The impact of Type 1/Type 2 error is likely, and it is not clear why schizophrenia should be inherently more complex. At present, we cannot resolve these possibilities.

## Public Health Implications

The public health importance of schizophrenia is clear, and the rationale for the search for genetic causes is strong. Schizophrenia research has never been easy: the current epoch of investigation into the genetics of schizophrenia provides a set of tantalizing clues, but definitive answers are not yet fully established. Findings from the accumulated literature appear to be more than chance yet sufficiently variable as to render “hard” replication elusive. The currently murky view of this literature may result from the competing filters of Type 1 and Type 2 error. The current literature could be a mix of true and false positive findings; however, it would be a momentous advance for the field if even one of the genes in Table 2 were a true positive result.

This body of work is not yet ready for wholesale translation into clinical practice. However, it is not premature to inform patients that this work is advancing and that it holds promise for new insights into etiology, pathophysiology, and treatment on the five- to ten-year horizon. On a larger scale, the treatment of the mentally ill mirrors the humanity of a society; in many societies, the return image is not flattering. If a specific genetic variation were proven to be causal to schizophrenia, this poor reflection might improve [28].

**GLOSSARY**

- Multiplex pedigree: A family grouping of genetically related individuals with multiple affected individuals.
- First-stage genome scan: An initial survey of the genome to identify regions that may contain genetic variants that could cause the disease under study. Subsequent stages focus on a smaller genomic region.
- Type 1 error: The probability of rejecting a true null hypothesis (akin to a false positive result).
- Type 2 error: The probability of accepting a false null hypothesis (akin to a false negative result).

## References

[pmed-0020212-b1] Saha S, Welham J, Chant D, McGrath J (2005). The epidemiology of schizophrenia. PLoS Med.

[pmed-0020212-b2] McGlashan TH (1988). A selective review of recent North American long-term followup studies of schizophrenia. Schizophr Bull.

[pmed-0020212-b3] Murray CJL, Lozpe AD (1996). The global burden of disease: A comprehensive assessment of mortality and disability from diseases, injuries, and risk factors in 1990 and projected to 2020.

[pmed-0020212-b4] Jeste DV, Gladsjo JA, Lindamer LA, Lacro JP (1996). Medical comorbidity in schizophrenia. Schizophr Bull.

[pmed-0020212-b5] Harris EC, Barraclough BB (1998). Excess mortality of mental disorder. Br J Psychiatry.

[pmed-0020212-b6] Murray RM, Jones PB, Susser E, van Os J, Cannon M (2003). The epidemiology of schizophrenia.

[pmed-0020212-b7] Mortensen PB, Pedersen CB, Westergaard T, Wohlfahrt J, Ewald H (1999). Effects of family history and place and season of birth on the risk of schizophrenia. N Engl J Med.

[pmed-0020212-b8] Plomin R, DeFries JC, Craig IW, McGuffin P (2003). Behavioral genetics in the postgenomic era, 3rd ed.

[pmed-0020212-b9] Sullivan PF, Kendler KS, Neale MC (2003). Schizophrenia as a complex trait: Evidence from a meta-analysis of twin studies. Arch Gen Psychiatry.

[pmed-0020212-b10] Sullivan PF, Owen MJ, ODonovan MC, Freedman RR, Lieberman J, Stroup T, Perkins D (2005). Textbook of schizophrenia. Genetics.

[pmed-0020212-b11] Korstanje R, Paigen B (2002). From QTL to gene: The harvest begins. Nat Genet.

[pmed-0020212-b12] Lewis CM, Levinson DF, Wise LH, DeLisi LE, Straub RE (2003). Genome scan meta-analysis of schizophrenia and bipolar disorder, part II: Schizophrenia. Am J Hum Genet.

[pmed-0020212-b13] Straub RE, MacLean CJ, ONeill FA, Burke J, Murphy B (1995). A potential vulnerability locus for schizophrenia on chromosome 6p24–22: Evidence for genetic heterogeneity. Nat Genet.

[pmed-0020212-b14] Schwab SG, Hallmayer J, Albus M, Lerer B, Eckstein GN (2000). A genome-wide autosomal screen for schizophrenia susceptibility loci in 71 families with affected siblings: Support for loci on chromosome 10p and 6. Mol Psychiatry.

[pmed-0020212-b15] Moises HW, Yang L, Kristbjarnarson H, Wiese C, Byerley W (1995). An international two-stage genome-wide search for schizophrenia susceptibility genes. Nat Genet.

[pmed-0020212-b16] Maziade M, Roy MA, Rouillard E, Bissonnette L, Fournier JP (2001). A search for specific and common susceptibility loci for schizophrenia and bipolar disorder: A linkage study in 13 target chromosomes. Mol Psychiatry.

[pmed-0020212-b17] Lindholm E, Ekholm B, Shaw S, Jalonen P, Johansson G (2001). A schizophrenia-susceptibility locus at 6q25, in one of the world's largest reported pedigrees. Am J Hum Genet.

[pmed-0020212-b18] Schizophrenia Linkage. Collaborative Group (1996). Additional support for schizophrenia linkage on chromosomes 6 and 8: A multicenter study. Schizophrenia Linkage Collaborative Group for Chromosomes 3, 6 and 8. Am J Med Genet.

[pmed-0020212-b19] Sullivan PF, Eaves LJ, Kendler KS, Neale MC (2001). Genetic case-control association studies in neuropsychiatry. Arch Gen Psychiatry.

[pmed-0020212-b20] Fan JB, Zhang CS, Gu NF, Li XW, Sun WW (2005). Catechol-O-methyltransferase gene Val/Met functional polymorphism and risk of schizophrenia: A large-scale association study plus meta-analysis. Biol Psychiatry.

[pmed-0020212-b21] Shifman S, Bronstein M, Sternfeld M, Pisante-Shalom A, Lev-Lehman E (2002). A highly significant association between a COMT haplotype and schizophrenia. Am J Hum Genet.

[pmed-0020212-b22] Florez JC, Hirschhorn J, Altshuler D (2003). The inherited basis of diabetes mellitus: Implications for the genetic analysis of complex traits. Annu Rev Genomics Hum Genet.

[pmed-0020212-b23] Klein RJ, Zeiss C, Chew EY, Tsai JY, Sackler RS (2005). Complement factor H polymorphism in age-related macular degeneration. Science.

[pmed-0020212-b24] Zareparsi S, Branham KE, Li M, Shah S, Klein RJ (2005). Strong association of the Y402H variant in complement factor H at 1q32 with susceptibility to age-related macular degeneration. Am J Hum Genet.

[pmed-0020212-b25] Hageman GS, Anderson DH, Johnson LV, Hancox LS, Taiber AJ (2005). From the cover: A common haplotype in the complement regulatory gene factor H (HF1/CFH) predisposes individuals to age-related macular degeneration. Proc Natl Acad Sci U S A.

[pmed-0020212-b26] Edwards AO, Ritter R, Abel KJ, Manning A, Panhuysen C (2005). Complement factor H polymorphism and age-related macular degeneration. Science.

[pmed-0020212-b27] Haines JL, Hauser MA, Schmidt S, Scott WK, Olson LM (2005). Complement factor H variant increases the risk of age-related macular degeneration. Science.

[pmed-0020212-b28] Braslow JT (1995). Effect of therapeutic innovation on perception of disease and the doctor-patient relationship: A history of general paralysis of the insane and malaria fever therapy, 1910–1950. Am J Psychiatry.

